# Boxer Underwear Incorporating Textile Moisture Sensor to Prevent Nocturnal Enuresis

**DOI:** 10.3390/s20123546

**Published:** 2020-06-23

**Authors:** Valentin Gaubert, Hayriye Gidik, Vladan Koncar

**Affiliations:** 1 GEnie et Matériaux TEXtiles (GEMTEX) Laboratory, F-59100 Roubaix, France; hayriye.gidik@yncrea.fr (H.G.); vladan.koncar@ensait.fr (V.K.); 2 École Nationale Supérieure des Arts et Industries Textiles (ENSAIT), F-59100 Roubaix, France; 3 Department of Hautes Etudes Ingénieur (HEI)-YNCREA, Lille Catholic University, F-59000 Lille, France; 4 Engineering Department, University of Lille, F-59650 Villeneuve d’Ascq, France

**Keywords:** textile moisture sensor, urine leakage sensor, enuresis alarm, urinary incontinence

## Abstract

Around 15% of children still wet their bed after five years old. Although bedwetting alarms have proven to be effective to achieve nighttime dryness, they are cumbersome so children could be reluctant to use them. Therefore, the moisture sensor and wire were made unobtrusive by seamlessly integrated them into fully textile underwear by using conductive yarns. Consequently, the alarm acceptability should be enhanced by improving children’s comfort. Three conductive textile metallic yarns, made of silver or stainless steel, were considered to fabricate the urine leakage sensor. Silver-plated-nylon yarn, which showed the highest electrical conductivity, outperformed the stainless-steel yarns regarding its ability to detect urine leakage as well as its detection speed. Furthermore, it was proven to withstand multiple urine soakings and the following machine-washings, even at high temperature (60 °C). However, the electrical current, necessary to detect the leakage, tends to corrode the silver. Therefore, the detection circuit was adapted. Eventually, the designed leakage sensor was seamlessly integrated into a child’s trunk underwear, into which a miniaturized alarm can be plugged. The resulting textile underwear aims at replacing the rigid alarm system currently available, hence improving the quality of life of enuretic children and help them achieving nighttime dryness.

## 1. Introduction

Enuresis, also called nocturnal enuresis (NE), is defined as intermittent incontinence in children over five years old while they are sleeping [1]. There is no consensus on the prevalence of enuresis as it varies according to the methodology, precise definition used, age group, sex, and population. Indeed, Wang et al. [2] reported a prevalence of 7.3% of primary nocturnal enuresis in Mainland China while 43% of Pakistani children suffered from NE according to Shah et al. [3]. Butler et al., who evaluated the prevalence of enuresis among children aged 7.5 years, reported that 15.5% of them were bedwetters [4]. On average, NE affects 15–20% of five-year-old children, 5% of 10-year-old children, and 1–2% of people aged 15 years and over [5]. It is more common for boys than girls [6]. NE can be subcategorized into Primary NE (PNE) when children have never achieved six months of continuously dry nights. Secondary nocturnal enuresis refers to children who have relapsed although they had attained at least six months of nighttime dryness. In addition, enuresis can be associated or not with other lower urinary tract symptoms. Mono-symptomatic enuresis concerns the children who report no bladder or voiding problems associated with their bedwetting; otherwise, it is non-monosymptomatic enuresis. Butler et al. proposed the three-system model to explain the causes of enuresis [7], which are not fully understood yet. According to their model, NE results of polyuria (overproduction of urine due to a lack of nocturnal vasopressin release) and/or nocturnal bladder overactivity coupled with an inability to arouse from sleep to bladder signals. The elevated sleep arousal threshold has been particularly investigated [8,9]. Although there is an annual spontaneous cure rate of 15% [10], living with enuresis often impacts negatively the quality of life of both the child and his parents. On the one hand, Jonsson Ring et al. found that children with enuresis have impaired self-esteem that affects their relationships with friends [11]. On the other hand, Roccella et al. revealed that parents of enuretic children show significantly higher stress level than those of typically developing children [12]. In addition, they tend to be more anxious-depressed. Therefore, several therapies exist: simple behavioral intervention [13], alarm therapy [14], medication [15], transcutaneous electrical nerve stimulation (TENS) [16], and laser acupuncture [17]. A recent systematic review and meta-analysis from Peng et al. [18] concluded that enuresis alarms offer a superior treatment response and a lower relapse rate in well-motivated children, confirming the findings of Glazener et al. [14] back in 2006. Hence, the European Association of Urology recommends alarms as first-line treatment [19]. Although the complex mechanism underpinning alarm effectiveness is not fully understood, they are believed to trigger conditioning effects on arousal [20] and/or increase bladder capacity. Mowrer [21] reported the first enuresis alarm, which was bed based. Nowadays, body-worn alarms have replaced the old bell-and-pad type. Nonetheless, the principle is the same: the first drops of urine are detected by a leakage sensor inside the child’s pajama, triggering a sound-emitting device, hooked on the child’s T-shirt. Hence the child is awakened by an audio signal and/or vibration to complete their micturition with dignity. However, both the leakage sensor and the alarm are cumbersome for the child. Furthermore, wires are located near the child’s neck that can cause safety risks if they get entangled during a troubled sleep. Therefore, this treatment can be rejected by them [22] or dropped before any improvement in their condition. Hence, there is a need to design more convenient device with better acceptability. Progress in smart materials have paved the way for embedding electronic into textile garments. As a result, bulky sensors can be replaced by unobtrusive ones that are comfortable to wear. These electronic textiles can be life-changing for many patients who need continuous monitoring. More specifically, they could significantly improve the life of enuretic children by unobtrusively, without causing any wearing discomfort, detecting the incontinence. In their review of smart fabrics sensors [23], Castano et al. reported textile moisture sensors made with stainless steel yarns [24] or PEDOT:PSS coating [25]. Some others are made with carbon nanotubes (CNT) [26]. Printed silver-ink [27,28] has also been reported for various applications such as monitoring garment micro-climate or moisture level in wound dressings [29]. More specifically, Parkova et al. proposed textile humidity sensors, embroidered [30] or woven [31], and specially designed to detect enuretic episodes. Nonetheless, neither their durability nor their potential embedding into a garment has been studied. Fernandes et al. reported a full system consisting of underwear integrating a wetness sensor and alarm [32]. However, it was designed to detect pad overflow in adults suffering from urinary incontinence. Thus, the comfort and acceptability of the moisture sensor have not been carefully taken into account since the subject was already wearing a pad below the underwear, thus protecting his skin. Briedis et al. [33] proposed a smart garment prototype for enuresis. Nonetheless, the sensors were embroidered on an existing garment, whereas in this study the sensors are simultaneously and seamlessly knitted inside the garment on an industrial knitting machine. Therefore, the resulting prototype can be massively industrialized. 

In Section 2, the underwear system is described. Then, the textile humidity sensors to detect urine leakage designed and fabricated with three different conductive yarns are discussed. Finally, the resulting underwear, incorporating the leakage sensor and integrating a miniaturized electronic alarm, is presented. In Section 3, the methods used to evaluate the performances of the sensors regarding their ability to detect the urinary leakage and the speed of such detection are described. In addition, as they are meant to be soiled with urine, these sensors have to withstand not only urine corrosion but also the washing cycles. Hence, the tests used to assess their withstanding to urine corrosion with and without a small electrical current as well as machine washing at 60 °C are described. The results are presented and discussed in the last part. 

## 2. Materials

### 2.1. Description of the System

**Figure 1:**
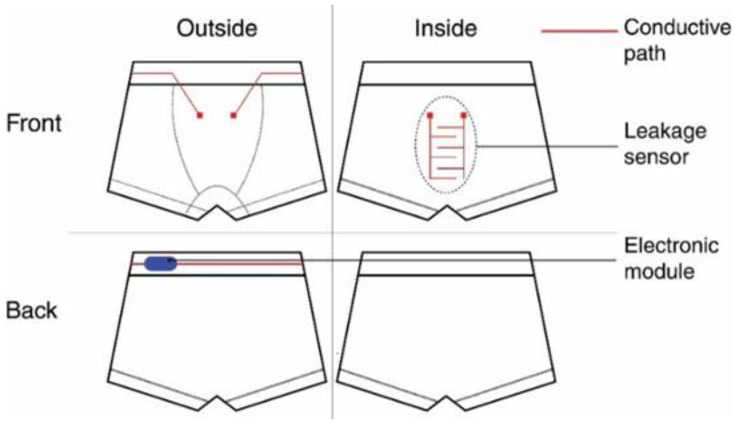
Schema of the system.

Figure 1 presents the schema of the system.

It is composed of two-layer underwear incorporating a textile leakage sensor inside, which is linked to an electronic module (blue) by textile conductive tracks (red). These tracks have been placed on the outer side to prevent any false positives, resulting from a short-circuit with the child’s skin. The inner and outer sides are connected by conductive platforms (small squares) that are stitched together with conductive yarn. The electronic module contains a simple processing unit and sound-emitting device, which is activated as soon as the sensor detects urine drops. The electronic circuit has been miniaturized to optimize the bulkiness of the module. The module is plugged with snap fasteners (male part on the module and female on the textile) at the back of the underwear. When the alarm is ringing, warning that incontinence has been detected, the child has to unplug the module to shut it down. Thus, it forces him to wake up and take back control of his bladder, before extensively wetting his bed. It also prevents the parents from forgetting to remove the module before putting the underwear inside the washing machine, which can destroy it.

### 2.2. Leakage Sensors

**Figure 2:**
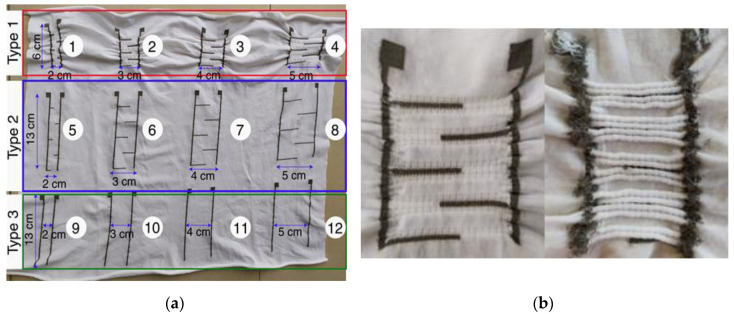
(**a**) Panel incorporating 12 sensors designs (Yarn A); and (**b**) detailed view of Type 1 sensors front and back sides.

Urine is easier to detect than sweat or air humidity as it has a much higher electrical conductivity because it contains several ions such as chloride (Cl^−^) or potassium (K^+^). Hence, urine leakage can be detected when there is an electrical connection, resulting from the contact with the conductive liquid, between a two-electrode sensor. To define the optimal pattern of those two electrodes, that enables the most sensitive urine detection, 12 different designs for sensors were tested. A panel including 12 sensors is shown in Figure 2a and a detailed image is presented for the Type 1 sensor in Figure 2b. The electrodes were made with conductive yarns, seamlessly knitted into a cotton/elastane substrate. Circular Seamless knitting is a technology developed by the Italian manufacturer Santoni that enables knitting a pattern of yarn X simultaneously with the textile substrate made of yarn Y. Consequently, the sensors are seamlessly integrated into the substrate, thus improving the comfort of the knitted structure. They were knitted on an industrial circular seamless knitting machine (SM8-EVO4J, Santoni (Brescia, Italy). Cotton was selected as substrate for its wettability and moisture management properties. Wettability is defined as the time in seconds for a drop of water to sink into the fabric. Cotton consists of cellulose which is hydrophilic compared to synthetic fibers such as polyester that are hydrophobic. Consequently, liquids are absorbed more rapidly by cotton than polyester. This behavior towards liquids is of prime concern as it determines the reactivity of the leakage sensor. Indeed, the higher are the wettability and capillary flow rate, the faster are the urine drops absorbed and spread out to close the detection circuit, formed by the two-electrode sensor, thus enabling a faster detection to wake the child up before too much urine has leaked. Furthermore, cotton is comfortable and hypo-allergenic which is all the more suitable for underwear. Besides, the cotton used is OEKO-TEX certified, which guarantees that it does not contain products that are toxic to the body or the environment. It seems mandatory for children underwear. 

A silver-plated-nylon yarn from Noble Biomaterials Inc. (Scranton, PA, USA), and two stainless steel yarns from Bekaert (Zwevegem, Belgium) (referred to as Yarns A–C, respectively) were tested. Table 1 gives their characteristics such as thickness, composition, and electrical resistance. It should be noted that there is a major difference between those yarns regarding their metallization. Yarn A is made of 36 filaments of polyamide entirely coated with silver that are twisted together. As a result, the entire surface of the yarn is continuously conductive. Yarns B and C consist of pure stainless-steel fibers that are twisted with the textile fibers which are not electrically conductive. Hence, the overall conductivity of Yarns B and C is determined by the contacts between stainless steel fibers among the textile fibers, resulting in a non-homogeneous linear and surface conductivity. This difference of structure accounts for the difference in the yarns’ conductivity although silver and stainless steel are both very good electrical conductors.

Two panels of 12 sensors, as shown in Figure 2a, were fabricated with each yarn. The following parameters were tested:

- The space between the two vertical lines, made of cotton fabric, which determines the sensor reactivity. Four different separating distances were tested (2, 3, 4, and 5 cm). The sensors consisting of only vertical lines are referred to as Type 3 sensor (see Figure 2a).
- The presence of horizontal lines which are supposed to improve the speed detection by reducing the gap between the two conductive electrodes. This type of sensors is referred to as Type 2 sensor (see Figure 2a).
- A more elaborate knitted structure was considered (see Figure 2b). Indeed, on the front side, it creates a sort of ditch, around the horizontal lines, which should improve the water absorption. At the backside, the flanges prevent any short circuit between horizontal tracks. Besides, the conductivity of the horizontals tracks is improved as the creases create extra contacts. This type of sensors is referred to as Type 1.

It should be noted that the line’s thickness, which is determined by the number of yarn loops, knitted with the conductive yarns, impacts the electrical conductivity. Indeed, the more conductive material there is, the more conductive is the track. It was fixed to four loops regarding a previous study that shows one or two loops are not sufficient. On the one hand, if the yarn is not very conductive, the resulting line is not able to detect or sense anything. On the other hand, it is too risky because the least damages (occurring during the manufacturing process or lifetime) can destroy the whole smart structure as the electrical continuity is irremediably lost. In addition, it should be borne in mind that conductive yarns are far more expensive than classical fibers so they should be used wisely. Consequently, a four-loop-line structure was selected to obtain reliable conductive tracks with a satisfying electrical conductivity.

### 2.3. Underwear Manufacturing

To be able to detect any leakage, the underwear has to be close-fitted. Hence, a trunk cut was selected. It was made with cotton, which has proved to be a suitable substrate for the sensor (see Section 2.2). Elastane was also needed to obtain the tight fit. The tube of fabric for the trunk was knitted on the same seamless circular knitting machine as the leakage sensor (SM8-EVO4J, Santoni). As it is an industrial knitting machine, the scale-up of the presented garment can be taken for granted. The tube was folded in two, resulting in a two-layer-garment (see Figure 1). The legs were cut then sewn.

**Figure 3:**
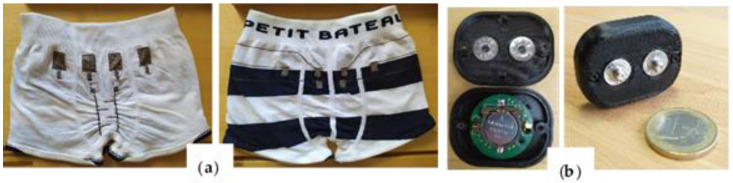
(**a**) Trunk integrating leakage sensor; and (**b**) photographs of the electronic module.

The manufactured trunk is presented in Figure 3a Snap fasteners were put at the back as children’s interviews revealed that most of them are used to sleeping on their belly. The female parts of the snap fasteners, made with stainless steel, were put with a press machine. Four squares are also visible above the leakage sensor but they should be not be confused with the leakage sensor. Their role and characteristics have been presented in another study.

Figure 3b shows the electronic module. It consists of a round printed circuit board, on which are mounted the two circuits (detection and alarm ones), enclosed in a tiny 3D printed box. The audio signaling device is a mini-buzzer that produces a sound level of up to 80 dB. More specifically, the sound is emitted at a low-frequency 500 Hz square wave tone, which showed the best performances in waking up children compared to other alarms (high-frequency and voice alarm) [34]. The electric power is supplied by a 3-V coin cell battery. The device stays in deep sleep mode until leakage is detected to optimize the power consumption. As a result, the battery is expected to last two months, after which most of the children are cured [35], with an alarm occurring every night. When the leakage is detected, the buzzer circuit is alimented until the child unplugs the electronic module, which stops the alarm following 30 s (explanations on this delay are given in Section 4.4).

## 3. Methods

### 3.1. Electrical Characterization of the Sensors

The electrical resistance of the conductive tracks (horizontal and vertical lines) of each type of sensors from each yarn was measured with a multimeter (VC276, Voltcraft (Hirschau, Germany)). The intra and extra resistance variabilities of the conductive lines were evaluated. For the intra-line resistance variability, 20 resistance measurements were done on the same line. For the extra-line resistance variability, the resistance was measured on each of the knitted lines of the two panels. More specifically, for each yarn, 128 measurements were done. As horizontal lines have different lengths, linear resistance (Ω/cm) was calculated.

### 3.2. Ability to Detect and Reactivity of the Patterns

**Figure 4:**
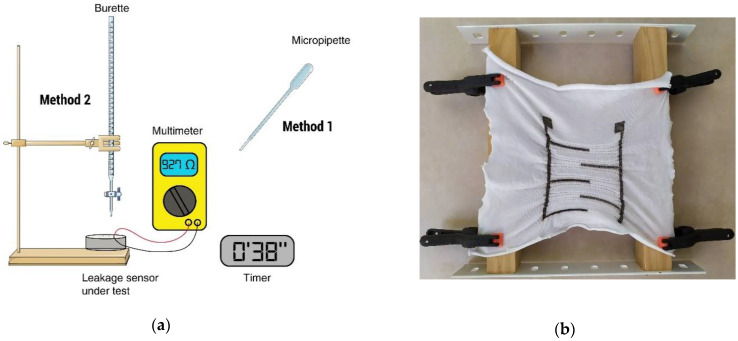
(**a**) Experimental Setup; and (**b**) sensor under test mounted on the frame.

The ability to detect leakage and the detection speed were evaluated for each sensor by using two methods. For both methods, a multimeter with a maximal range of 107 ohms was connected to the sensors platform to record the resistance. Leakage is considered detected once the multimeter can detect a resistance between the two platforms, meaning that urine has shortened the detection circuit. The detection speed was recorded by a timer (see Figure 4a). The textile leakage sensor was mounted on a hand-made frame (see Figure 4b).

- Method 1 consists in pouring five drops of artificial urine, with a micropipette, at the top of the sensors (between the higher horizontal lines) and record the detection speed if the sensors can detect leakage. Originally, the conductive liquid was supposed to be placed in the middle of the sensors but stainless-steel sensors showed very poor results, thus it was changed to the top.
- Method 2 consists in continuously pouring drops of artificial urine, at a constant flow, with a burette until leakage is detected.

Before each measuring session, the panels were conditioned for 4 h into a climate chamber (HCP153230V, Memmert GmbH (Schwabach, Germany)) at 20 °C with a relative humidity of 65% to ensure reproducibility. They were systematically rinsed with tap water, and then line dried after each session.

Although detailed recipes of artificial urine can be found in the literature, only the overall electrical conductivity matters for its detection by the designed resistive sensor. Therefore, alimentary salt, containing sodium chloride (NaCl), was dissolved in demineralized water to obtain artificial urine. Different mean values of urine conductivity have been reported (from 1.75 S·m^−1^ [36] to 2.15 S·m^−1^ [37]) since urine presents intra- and extra-subject variability [38]. The lowest reported conductivity (1.75 S·m^−1^) was chosen to ensure the reliability of the sensor. The quantity of salt (NaCl) needed to be added into demineralized water was calculated using Equations (1) and (2).

σ = λ_Cl−_·[Cl^−^] + λ_Na+_·[Na^+^] = (λ_Cl−_ + λ_Na+_)·x (1)

where σ is the conductivity of the artificial urine (1.75 S·m^−1^), λ_Cl−_ is the molar conductivity of Cl^−^ (7.63 × 10^−3^ S·m^2^·mol^−1^), λ_Na+_ is the molar conductivity of Na^+^ (5.01 × 10^−3^ S·m^2^·mol^−1^), and x is the molar concentration of NaCl (mol·m^−3^).

y = M(NaCl)·x(2)

where y is the weight of NaCl (g·m^−3^), M(NaCl) is the molar mass of NaCl (58.44 g·mol^−1^), and x is the molar concentration of NaCl (138 mol·m^−3^). The weight of salt needed (called y) is 8091 g·m^−3^ or 8.091 g·L^−1^. Consequently, 8.1 g of alimentary salt (Les Salins du Midi (Aigues-Mortes, France)) was dissolved into 1 L of demineralized water.

For each of the 12 designs, five and three leakages, respectively, were simulated for Methods 1 and 2. The following speed detection was recorded. Besides, the circuit resistance, when closed with the artificial urine, was measured once. It was measured 3 min after the leakage was detected since the resistance tends to decrease for a short while after the detection as the artificial urine continues to spread, thus contacting more surface of the sensor.

### 3.3. Withstanding to Machine Washing

As the sensor is intended to be soiled with urine, it must be washed. Since the bed sheets are also soiled, the parents usually put the soiled underwear with them in the washing machine. Hand washing is hardly acceptable for parents, especially in the middle of the night. A previous study [39] has already investigated the withstanding of this silver yarn to machine washing with the delicate cycle at 30 °C. However, some parents would rather use higher temperature such as 60 °C to clean urine soiling. Consequently, the withstanding of a machine washing at 60 °C was investigated. A domestic washing machine (ENF 1486 EHW, Electrolux (Stockholm, Sweden)) was used. Following the findings of our previous study, a liquid detergent (Rainett (Mainz, Germany)), which does not contain bleaching agents, was preferred to powder ones, as they tend to wipe out the conductive layer, thus reducing the yarn’s electrical conductivity. For every washing cycle, 1.8 kg of fabrics were put inside the machine with the samples to simulate real household washing, in compliance with the AATCC135 standard for laundry. Only white fabrics were used as ballast in order not to accidentally stain the sensor. Indeed, as metallic yarns in contact with water have been reported to produce red-brown deposition in some cases [40]; this possible phenomenon should not be confused with a fabric discoloration. Three leakage sensors (2, 6, and 10 (see Figure 2a)) of each yarn were washed 20 times in the described conditions. The conductivity of the lines (vertical and horizontal) was measured after every five cycles. In addition, the speed detection was compared to the reference ones (without washes).

### 3.4. Corrosion Resistance to Urine

As the designed leakage sensor is intended to be soiled with urine, containing many organic components such as urea and inorganic ions, it has to be chemically inert. Otherwise, the metal part of the sensors could be oxidized or corroded, thus impairing its ability to detect leakage. Indeed, urea solution and cattle urine have been reported to corrode steels [41]. Consequently, the sensor’s ability to withstand urine corrosion was tested to ensure its durability. Since no textile standards, concerning the corrosion resistance to urine, have been found, a method is proposed. Two sensors of each yarn (more specifically, Sensors 4 and 9 (see Figure 2a)) were extensively soaked with real urine and abandoned three consecutive times for 20 h periods. The three urine samples were provided by one consenting adult on different days. Twenty hours is estimated to represent almost 60 use cycles of the system. Indeed, parents from enuretic children reported washing the soiled textiles (underwear, bedsheets) directly after the leakage, before going back to bed. As a result, an approximate period of 20 min takes place between the underwear’s soiling with urine and its cleaning. Nonetheless, it could be forgotten to wash it. Hence, the resistance to urine corrosion was evaluated through three consecutive 20-h periods. After each period, the sensors were abundantly rinsed with tap water and line dried before their resistance was measured. The visual aspect, as well as the linear resistance of the conductive lines, were compared between the reference and soaked samples. 

### 3.5. Corrosion Resistance to Urine When an Electrical Current Is Flowing

As the leakage detection is based on an electrical circuit which is closed by the leakage itself, an electrical current will flow in the detection circuit. Although this current is weak (<0.3 mA which is below the human perception), it will generate directional ions movement, thus accelerating the corrosion process. Once wet, the detection circuit can be seen as an electrolytic cell. The two electrodes become the anode and the cathode and the wet substrate the electrolyte. The chemical reactions occurring at the cathode and anode can potentially impair the electrical conductivity of the electrode if the metal is reduced or oxidized in a less conductive element. In addition, the potential migration of metallic ions, across the substrate, can stain it, which is not acceptable. Therefore, the resistance to corrosion, when the electrical current is flowing, was tested for three yarns. A textile moisture sensor (Sensor 3 (see Figure 2a)) of each yarn was soaked with artificial urine and then connected to 3-V battery for different periods (1, 5, 10, 30, and 60 min). A 3-V battery was selected as it is the power source of the electronic module. After each period, the sensor was rinsed and line dried before the resistance measurement. The anode (electrode connected to the positive pole) and the cathode (negative pole) were measured separately as different chemical reactions took place. Moreover, the visual aspect of the leakage sensor was examined.

### 3.6. Sensor’s Performances under Different Wearing Conditions

**Figure 5:**
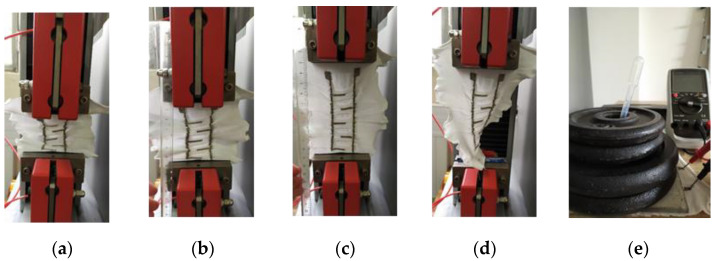
(**a**) bent sensor; (**b**) normal sensor; (**c**) elongated sensor (50%); (**d**) twisted sensor; and (**e**) sensor under a load of 6 kg.

As the moisture sensor is integrated into boxer underwear, it should work under different wearing conditions that can degrade its performances. Hence, such conditions were reproduced to evaluate their impact. More specifically, when worn by a child at night, the sensor can be subjected to mechanical and thermal stresses. Regarding mechanical stress, the sensor can be stretched, twisted, or bent when the child moves. Therefore, an M250-2.5 testing machine (Testometric Co. Ltd. (Rochdale, United Kingdom)) was used to reproduce those conditions and measure the resulting speed detection of the sensor:

Bending: The sensor was bent (see Figure 5a).
Stretching: The sensor was elongated by ΔL/L = 50% and 100% (see Figure 5c).
Twisting: The sensor was twisted with an angle of θ = 90° (see Figure 5d). It was also elongated for the photo but was not for the test.

In addition, if the child sleeps on their belly, his pelvis applies a stress force on the sensor. Therefore, the sensor’s performances were evaluated when a 7 kg load, representing the child’s pelvis weight, was put over it. The sensor was isolated from the metallic loads by a polycarbonate sheet. A small hole (4 mm diameter) was drilled in the sheet to pour the artificial urine. Concerning the thermal stress, the relative humidity (RH) of the sensor’s microenvironment could be the main challenging parameter. Indeed, it can vary significantly depending on the season and on what the child is wearing above the underwear, which can make him sweat. Therefore, the sensor was conditioned at 20 °C with two relative humidity levels (35% and 95%) in the climate chamber.

## 4. Results and Discussion

### 4.1. Electrical Properties

Table 2 shows the electrical linear resistance of the different conductive lines.

**Figure 6:**
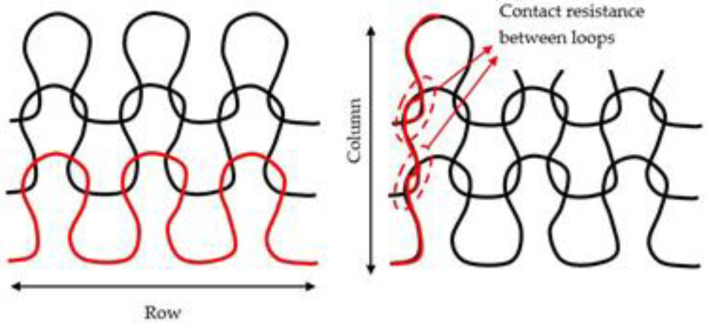
Electrical pathways in the knitted structure.

It is noticeable that the lines made of silver yarn are much more electrically conductive than those of stainless steel (few ohms/cm compared to 107 ohm/cm). It turns out that the conductivity of Yarns B and C is so low that the resistance of the entire line (13 cm) was out of the multimeter range. Consequently, the electrical resistance was measured on a few centimeters (3 cm for Yarn B and 1 cm for Yarn C) and presented in Ω/cm. Yarn C was finally discarded from the study as no sensor made with it could detect urine leakage (see Section 4.2). Such high resistance magnitudes measured with stainless steel seem surprising regarding the electrical resistance of the yarns given by the suppliers (see Table 1). Although the silver yarn is intrinsically more conductive, it is in the same order of magnitude. The structure of the stainless-steel yarn, detailed in Section 2.2, can account for these high results. Indeed, the yarns have been made conductive by blending cotton or polyester with stainless-steel fibers and twisting them together very tightly in order to ensure some contacts between the stainless-steel fibers. Hence, electrical conductivity can be obtained all along the yarn. However, in the presented structures, these yarns were knitted to interlace together with loops (see Figure 5). The overall electrical conductivity of the knitted line results from the contacts between metallic fibers of neighboring loops. As the stainless-steel yarns are mostly composed of textile fibers (cotton or polyester), the metallic fibers of yarns are most likely to be in contact with textile fibers than another metallic fiber inside the loops. Consequently, the electrical resistance of a knitted structure is much higher than the yarn itself. It is even more important for rows than columns. Indeed, the electrical conductivity of a knitted structure is anisotropic. As illustrated in Figure 6, the electrical current runs along yarns on rows. Consequently, the resistance measured on rows is relatively representative of the yarn’s one. On the contrary, the current has to flow from one loop to another in columns. In the case of this stainless-steel yarn, the contact resistance between loops is high as stainless-steel fibers, thus they are not very likely to touch each other. This accounts for the significant difference obtained between vertical and horizontal lines made with stainless steel yarns. Comparing to vertical resistance (107 Ω/cm), the horizontal resistance is 103 and 104 Ω/cm for Yarns B and C, respectively. It seems not to be the case with silver yarn, even the opposite regarding Table 2. However, the figures are misleading because the width of the horizontal lines is smaller than the vertical ones, which accounts for their higher resistance. Indeed, as illustrated in Figure 5, the width of loops is larger than their length (the three loops in a row are longer than the three loops in columns).

The resistance of Type 2 and 3 vertical lines is smaller than those of Type 1 because 1 cm actually represents 2 cm that is pleated. Nonetheless, Type 1’s vertical resistance is not exactly two times higher than Types 2 and 3, as extra contacts are made by the folds. These contacts are random, which results in higher standard deviation. Horizontal lines resistance of Type 1 is slightly lower than Type 2, probably because the lines are more structured by the construction. The variability of silver yarn resistance is relatively low, confirming the homogeneity of the conductive silver layer covering those yarns, compared to the standard deviation obtained with the stainless-steel yarns (B and C), illustrating the random contacts between metallic fibers. Vertical lines’ resistance of stainless yarn was no longer measured regarding the high standard deviation, which makes any conclusions difficult.

### 4.2. Ability and Reactivity of the Sensors

As a reminder, the pattern design numbers are shown in Figure 2a. Table 3 shows the urine detection speed of each pattern with the two methods. It should be noted that the liquid drops were poured at the top of the sensor between the first horizontal lines of the comb (see Figure 4b) because the stainless-steel sensor cannot detect them otherwise. The cases are left blank when the leakage was not detected.

Only Type 1 sensors were able to detect every leakage no matter the metallic yarn and the method used. For Type 2, all the sensors made of silver were able to detect the five liquid drops poured in Method 1, whereas only Sensor 5 of stainless steel was able to detect them correctly. Sensor 6 sensed only two out of five leakages after a long delay. 

Few Type 3 sensors were able to detect the five drops because they were absorbed by the cotton substrate before attaining the conductive vertical lines. It highlights the role of the substrate in the sensor that should be carefully considered when designing a humidity sensor. To overcome the substrate absorption, Method 2 was used. It consists of continuously pouring drops, at a constant flow, until leakage is detected. All the sensors were able to detect the simulated leakage with this method. Besides, the detection was globally faster as the wetted area is proportional to the volume poured. Indeed, Kawase et al. [42] found that the kinetics model, presented by Kissa et al. [43] (given by Equation (3)), of liquid spreading on impermeable fabrics, holds also for water in cotton fabric, provided that the coefficient n is much smaller (0.15) than the theoretical value (0.33) proposed by Kissa.

A = K (γ/η)^u^·V^m^ (3)

where A is the area covered by the spreading liquid, K is the coefficient dependent on the advancing contact angle of the liquid on the fibers, the permeability, and thickness of the fabric, t is the spreading time, γ is the surface tension, η is the viscosity, and V is the volume of the liquid.

Consequently, if A is the area between the two electrodes, the spreading time needed to reach them decreases by increasing the volume of the conductive liquid poured. Although all Type 3 sensors proved to be able to detect the leakage, the delay is too long to prevent bedwetting as the child’s micturition is much probably completed after 20 s. As a result, Type 3 sensor pattern was found not suitable for leakage sensors. On average, Type 2 sensors give a better performance with reduced detection time. Thanks to the horizontal lines, the detection speed does not depend on the distance between the electrodes so a sensor with a wider detecting zone (for example, Sensor 8) has similar performances as shorter ones (for example, Sensor 6). However, detection time longer than 10 s is not suitable. Therefore, Type 1 sensors are preferred to the two other types.

The detection area of Sensor 1 is too limited and it is highly prone to false positives as the conductive lines are very close to each other. Sensor 4 is slightly too wide to be integrated into a five-year-old’s trunk underwear. Finally, Sensor 3 was selected rather than Sensor 2 to ensure a wider detection zone. 

**Figure 7:**
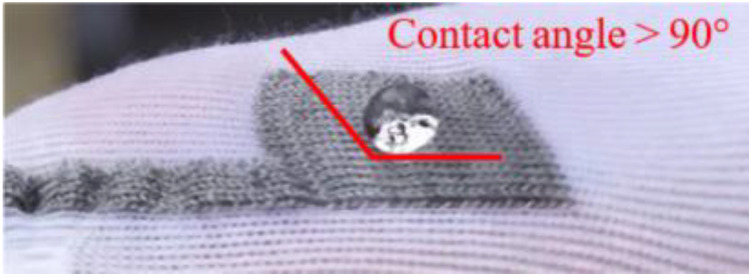
Hydrophobic behavior of Yarn B.

For Method 1, silver was able to detect more leakages than stainless steel (10 leakages compared to 7 leakages). Sensors 7 and 8 made with stainless steel were not able to detect leakage because too little conductive liquid was in contact with the yarn. It was also the case for Sensor 10. It was found that the stainless-steel line resistance decreases when wet (as the liquid is more conductive than the sensor itself), thus these sensors were adapted to detect heavier leakage. Nonetheless, for both methods, the silver sensors’ detection speed is faster than stainless steel. To account for such difference, it should be reminded that the stainless-steel fibers are mixed with hydrophobic polyester ones. Figure 7 shows that the contact angle between the drop and the fabric is superior to 90°. Consequently, very few stainless-steel fibers are in contact with the urine drop until the yarn absorbs it, which impedes detection. Although the silver-plated-nylon is hydrophobic too, the urine does not need to be absorbed by the yarn to be in full contact with the conductive material as silver covers every filament. As a result, even if the artificial urine arrives at the same time in the vicinity of electrodes, whatever their material (silver or stainless steel), the stainless-steel ones need to absorb the urine to be able to detect it. Thus, delaying its detection. Moreover, stainless steel is much less electrically conductive than silver.

Table 4 shows the short circuit resistance of the sensors. For the silver sensors, it was in the range of 103 ohms which is significantly lower than the stainless-steel ones (107 ohms). Such a high resistance is comparable to the skin resistance which can result in many false positives. For example, simple manipulations of the electronic module can trigger the alarm if the two metallic connectors are in contact with the hand skin. Regarding all the considerations listed above, none of the stainless-steel yarns tested seem suitable to fabricate the textile leakage sensor.

### 4.3. Withstanding to Washing

As the soiled underwear will have to be machine washed several times, its withstanding of a washing machine at 60 °C with a bleach-free detergent was tested. Table 5 shows the electrical resistance evolution with the washing cycles, until 20 washing cycles.

Even if the electrical resistance of the silver yarn electrodes tends to increase with the washing cycles, it is so conductive that this increase does not impact its capacity. Indeed, the sample washed 20 times shows the same ability and reactivity to detect urine leakage. As a result, this yarn is suitable for a leakage detector, providing bleach-free detergent is used for the washing. Indeed, it has been shown that powder detergents can deeply impact silver-plated yarns. Stainless steel is much more impacted by the washing process. The electrical conductivity was multiplied by 10 in only 20 washings. The capacity of the leakage sensor that was originally relatively poor became even poorer. Leakage cannot be detected every time with the sample washed 20 times. When detected, the delay is so long that the child’s micturition may be complete. This yarn seems not suitable either regarding its withstanding of the washing machine.

### 4.4. Corrosion Resistance to Urine

The corrosion resistance of silver Sensors 4 and 9 was evaluated to ensure their durability in view of integrating one of them in boxer underwear for an enuretic child. In addition, Sensor 3 made with stainless steel was tested to compare with the silver one. Table 6 shows the corrosion resistance of the sensors to urine. Visually, no difference can be observed between the soiled samples and the reference one. 

Urine soiling impacts the electrical resistance of the silver-yarn electrodes, which was almost multiplied by three after being exposed 60 h to urine. Stainless steel seems less impacted as its resistance less than doubled. For both yarns, this damage seems less significant in the third exposure period (from 40 h to 60 h). It should be kept in mind that urine has an intra-subject variability so it was not the same for all three periods. It can account for this observation. Although the electrical conductivity of the different lines soiled 60 h decreased, it was still acceptable. Indeed, a leakage could be detected with the two pouring methods as quickly as recorded in the previous part. 

### 4.5. Corrosion Resistance to Urine When Submitted to an Electrical Current

Stainless steel seems to be less impacted by the flowing current than silver. However, it should be kept in mind that, due to its higher resistance, the short circuit resistance of the sensor is also high. Hence, the current intensity is very weak (a few µA). The silver sensor is significantly damaged when current is running, more specifically the horizontal lines of the anode.

Horizontal lines are closer to the cathode so chemical reactions are most likely to take place at this location. Indeed, after only 5 min, the resistance was almost multiplied by 15. After 10 min, it exceeded the multimeter range (>107 ohms), impacting the sensor performance. As the horizontal lines were no longer electrically conductive, the sensor took around 12 s to detect the leakage, similar to the Type 2 sensors. After an hour neither the horizontal nor the vertical lines of the anode were still conductive. Hence, the leakage sensor was no longer functional. Unlike the anode, the cathode resistances (vertical and horizontal) were barely impacted by the electric current. Moreover, visually, the anode was distinguishable from the cathode because the bright grey darkened and there was a yellowish halo around the electrode platform. An electrochemical reaction turning pure silver into a less conductive and darker element took place at the anode. Most probably, it is the formation and reduction of an AgCl film on the Ag substrate, which was documented by Birss et al. [44], according to Reaction (1).

Ag (s) + Cl^−^ → AgCl + e^−^ (4)

However, as urine contains many elements, one more complex or even several chemical reactions could account for the presented observations. Nonetheless, it is not the purpose of this study. 

Although an electrical current is necessary to detect the leakage, it should run in the circuit at little as possible to prevent this reaction. Originally, the current was supposed to flow until the electronic module, which should be unplugged by the child. However, between the short circuit, that triggers the alarm, and its removal, it takes 15 s at least. Indeed, enuretic children are known to be deep sleepers [9]. As shown in Table 7, the current should not flow more than 5 min, which corresponds to a lifespan of 20 cycles in the best-case scenario. After 5 min, the sensor can no longer be considered reliable. Such a short lifetime is not acceptable for users as the children are most likely to wet their underwear more than 20 times before achieving dry nights. Although on a regular basis the set is composed of two or three underwear pants with one electronic module, a solution to overcome the reported issue must be found. Therefore, the electronic part of the system was modified. Instead of controlling the sensor and the alarm with the same circuit, two different circuits were designed. Hence, as soon as the detection circuit is closed by the incontinence, the current switches to the alarm circuit to power the buzzer. As a result, the current will flow only a few seconds (maximum 3 s) through the sensors, thus expanding its lifespan up to 60 cycles. Nonetheless, it is the withdrawal of the electronic module by the child that should stop the alarm. To detect this withdrawal, small electrical impulses are injected into the detection circuit every 30 s. If no current can flow, meaning the module has been withdrawn, the alarm is shut down.

### 4.6. Sensor’s Performances under Different Wearing Conditions

**Figure 8:**
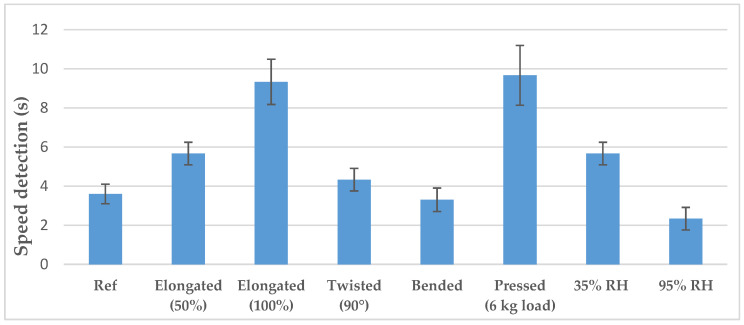
Sensor’s performances under different reproduced wearing conditions.

Sensor 3 was used for these tests. In addition, the speed detection of Sensor 3, without elongation and conditioned at a relative humidity of 65%, was taken as reference. Figure 8 shows the sensor’s speed detection under the conditions described in Section 3.5. When elongated, the detection speed is higher than the reference one as the path between the electrodes is longer. The increase is proportional to the percentage of elongation. Neither bending nor twisting had a significant impact on the detection speed as the path between the electrodes did not vary significantly. When pressed homogeneously with a 6 kg load, reproducing the child’s pelvis when a child sleeps on their belly, the detection speed was twice the reference one. This can be explained by the fact that the liquid is forced to move through the substrate, whereas it can spread on its surface without the load. Although the sensor performances could be slightly degraded when the child sleeps on the belly, the sensor is still functional.

Regarding the relative humidity, the wetter is the substrate, the faster is the detection. Even at 95% humidity, the sensor is not short-circuited, which could have resulted in false-positive due to child’s perspiration. Furthermore, to avoid false detection with sweat, the sensor’s detecting threshold is based on the short circuit resistance with urine, which is lower than with sweat. Indeed, the electrical conductivities of sweat reported are much lower than that of urine (0.3 S/m in [45] and 0.556 S/m in [46] for sweat compared to 2.15 S/m in [37] for urine).

Silver sensors showed far better performances than the stainless-steel ones. Indeed, even though they were both able to detect some leakages (see Section 2.2), it was the case because the drops were poured close to the platform. Otherwise, the stainless-steel sensors were unable to sense urine. Consequently, the silver yarn was selected as the most suitable material to fabricate textile leakage sensors. Sensor 3 is thought to be the most adapted for a five-year-old’s underwear. Therefore, it was integrated into textile underwear to obtain a smart structure preventing bedwetting.

## 5. Conclusions

Although alarm intervention is known to be the most effective therapy for enuresis, alarm devices suffer from poor child acceptability because of their bulkiness. Therefore, a more convenient system, overcoming the limitations of the currently available ones, is presented. An unobtrusive textile urine leakage sensor was designed to replace classic rigid sensors that should be clipped inside the children underwear. The wire, linking the sensor to the alarm, in which the children could be entangled, was also seamlessly integrated into textile underwear. Twelve different sensor designs were knitted with three conductive textile yarns made of silver or stainless steel. Yarn C, made of 80% cotton and 20% stainless steel, was discarded since none of the twelve sensors made with it were able to detect a leakage correctly. Silver-plated-nylon yarn presents the highest electrical conductivity, resulting in better performance detection than Yarn B, made of 30% stainless and 70% polyester. Indeed, the sensors made with silver can detect liquid drops no matter their position, unlike stainless steel which can detect only at top of the sensor. Furthermore, the detection with silver is faster as it is more electrically conductive. Besides, polyester is hydrophobic which tends to repel the liquid from the sensing stainless fibers. As a result, silver was selected as the most suitable material for the textile leakage sensor. It could withstand extended urine soiling as well as 20 washing cycles in a machine at 60 °C with liquid detergent. However, a problematic corrosion phenomenon was highlighted when the sensor was submitted to an electrical current. It was found that current should not flow more than 5 min, otherwise the sensor performance is significantly degraded. Therefore, the electronic part was adapted to limit as much as possible the current in the detection circuit. Hence, the sensor’s lifespan was extended to up to 30 cycles, which is thought to be sufficient as the children will have a set composed of at least two trunks. The urine leakage sensor as well as the conductive tracks, to connect an electronic module, were incorporated seamlessly into these trunks. Hence, the comfort, thus the system’s acceptability by the children, should be significantly enhanced. The presented electronic textile underwear should improve the quality of life of enuretic children and help them achieve nighttime dryness, which is a milestone in their development. This will be evaluated in a further study consisting in testing the presented smart structures on a cohort of enuretic children. In addition, the textile unobtrusive leakage sensor could be life-changing for adults suffering from incontinence.

## Figures and Tables

**Table 1:** Yarns’ characteristics.

Yarn	Metal	Thickness	Composition	Resistance	Reference
A	Silver (Ag)	78 dtex/34 f	Silver 20%/Polyamide 80%	<6 Ω/cm	30700341W
B	Stainless Steel (SS)	200 dtex	Stainless Steel 30%/	25 Ω/cm	9002676
			Polyester 70%		
C	Stainless Steel (SS)	200 dtex	Stainless Steel 20%/	40 Ω/cm	9031166
			Cotton 80%		

**Table 2:** The electrical linear resistance of the sensors.

		Electrical Linear Resistance (Ω/cm)							
		Vertical Lines				Horizontal Lines			
		Type 1	SD	Types 2/3	SD	Type 1	SD	Type 2	SD
**Yarn A**	Extra	**3.58**	0.26	**2.63**	0.16	**5.08**	0.42	**5.51**	0.96
**(Silver)**	Intra	**3.29**	0.17	**2.61**	0.06	**4.45**	0.15	**4.85**	0.15
**Yarn B**	Extra	**4.06 × 10^7^**	5.75 × 10^6^	**3.74 × 10^7^**	8.51 × 10^6^	**8.26 × 10^2^**	2.75 × 10^2^	**1.30 × 10^3^**	6.73 × 10^2^
**(SS1)**	Intra	**3.09 × 10^7^**	7.60 × 10^6^	**3.68 × 10^7^**	7.72 × 10^6^	**1.08 × 10^3^**	3.73 × 10^2^	**9.85 × 10^2^**	2.75 × 10^2^
**Yarn C**	Extra	**4.14 × 10^7^**	8.36 × 10^6^	**4.55 × 10^7^**	1.14 × 10^7^	**6.22 × 10^4^**	8.98 × 10^4^	**4.21 × 10^4^**	2.13 × 10^4^
**(SS2)**	Intra	**3.87 × 10^7^**	1.03 × 10^7^	**4.53 × 10^7^**	1.51 × 10^7^	**5.69 × 10^4^**	1.66 × 10^4^	**7.03 × 10^4^**	9.98 × 10^3^

**Table 3:** Detection speed of the sensors.

		Detection Speed (s)											
		Type 1 (Tight Comb)				Type 2 (Comb)				Type 3 (Lines)			
		**1**	**2**	**3**	**4**	**5**	**6**	**7**	**8**	**9**	**10**	**11**	**12**
M1	**Ag**	**2.4** ± 0.5	**3.0** ± 0.7	**3.6** ± 0.5	**3.7** ± 1.3	**6.0** ± 2.1	**13.4** ± 5.2	**14.2** ± 8.0	**24.0** ± 10.7	**6.0** ± 2.5	**32.8** ± 12.0	**-**	**-**
	**SS**	**7.2** ± 1.5	**9.2** ± 3.4	**6.6** ± 1.9	**6.0** ± 2.5	**12.6** ± 3.7	**91.8** ± 75.3	**-**	**-**	**12.4** ± 4.8	**-**	**-**	**-**
M2	**Ag**	**2.0**	**2.3** ± 0.6	**3.0**	**3.3** ± 0.6	**6.3** ± 0.6	**11.6** ± 0.6	**11.6** ± 0.6	**12.3** ± 1.5	**6.3** ± 0.6	**13.3** ± 3.5	**22.6** ± 6.5	**30.6** ± 11.5
	**SS**	**5.6** ± 1.5	**6** ± 2.0	**5.6** ± 1.5	**5.6** ± 2.1	**9.7** ± 2.0	**19.6** ± 10	**25.3** ± 14.6	**23** ± 9.8	**8.3** ± 1.5	**18.3** ± 7.0	**30.3** ± 13.6	**42** ± 26.0

**Table 4:** Short-circuit resistance of the sensors.

	Resistance (Ω)											
	Type 1 (Tight Comb)				Type 2 (Comb)				Type 3 (Lines)			
	**1**	**2**	**3**	**4**	**5**	**6**	**7**	**8**	**9**	**10**	**11**	**12**
**Ag**	1.2 × 10^3^	1.5 × 10^3^	1.6 × 10^3^	1.9 × 10^3^	2.0 × 10^3^	2.8 × 10^3^	2.5 × 10^3^	3.2 × 10^3^	1.9 × 10^3^	1.0 × 10^4^	1.3 × 10^4^	1.52 × 10^4^
**SS**	2.5 × 10^7^	3.5 × 10^7^	4.0 × 10^7^	4.2 × 10^7^	3.3 × 10^7^	4.6 × 10^7^	2.5 × 10^7^	5.0 × 10^7^	2.9 × 10^7^	4.8 × 10^7^	5.9 × 10^7^	6.36 × 10^7^

**Table 5:** Withstanding of washing cycles.

	Silver Yarn				Stainless Steel			
	Vertical Lines		Horizontal Lines		Horizontal Lines			
	Type 1	Type 2/3	Type 1	Type 2/3	Type 1	SD	Type 2/3	SD
Réf	**19.7** ± 0.7	**31.6** ± 0.9	**11.1** ± 1.6	**12.1** ± 1.2	**1.08** **× 10^3^**	4.25 × 10^2^	**9.80** **× 10^2^**	3.32 × 10^2^
5 washes	**27.7** ± 1.0	**31.9** ± 1.5	**10.6** ± 3.2	**9.3** ± 1.5	**1.89** **× 10^3^**	9.23 × 10^2^	**1.60** **× 10^4^**	1.55 × 10^4^
10 washes	**22.8** ± 1.7	**30.9** ± 2.1	**10.8** ± 2.9	**7.8** ± 1.5	**7.69** **× 10^3^**	8.66 × 10^3^	**2.47** **× 10^4^**	1.80 × 10^4^
15 washes	**31.9** ± 2.1	**38.2** ± 3.5	**11.5** ± 4.3	**9.5** ± 1.8	**1.14** **× 10^4^**	4.37 × 10^3^	**4.55** **× 10^4^**	2.56 × 10^4^
20 washes	**29.2** ± 1.9	**39.5** ± 3.6	**14.4** ± 4.6	**9.9** ± 2.3	**1.06** **× 10^4^**	1.76 × 10^4^	**6.69** **× 10^4^**	2.35 × 10^4^

**Table 6:** Corrosion resistance to urine of the sensor.

			Resistance (Ω)			
	Lines	Sensor	Ref	20 h	40 h	60 h
Ag	Vertical	9	**31.0** ± 0.51	**47.1** ± 0.68	**71.1** ± 0.45	**81.8** ± 1.84
		4	**21.4** ± 1.84	**41.5** ± 0.64	**64.2** ± 0.96	**74.1** ± 2.24
	Horizontal	4	**14.3** ± 1.05	**21.4** ± 1.68	**27.6** ± 1.80	**32.3** ± 1.44
SS		4	**726.2** ± 134.5	**878.5** ± 79.5	**1140** ± 271.6	**1159** ± 224.1

**Table 7:** Corrosion resistance to urine under electrical current.

	Silver				Stainless Steel	
	Resistance (Ω)					
	Vertical Lines		Horizontal Lines			
Time (min)	+	-	+	-	+	-
0 (ref)	**19.7** ± 1.0	**20.2** ± 0.8	**14.6** ± 0.6	**13.4** ± 0.5	**604** ± 104	**622** ± 172
1	**41.8** ± 0.8	**34.6** ± 0.7	**29.9** ± 6.9	**16.01** ± 1.1	**571** ± 221	**703** ± 124
2	**46.3** ± 1.2	**32.4** ± 0.9	**37.6** ± 5.3	**18.3** ± 1.9		
3	**63.7** ± 1.6	**31.4** ± 0.9	**75.43** ± 18.2	**20.6** ± 1.3		
5	**72.4** ± 2.6	**38.88** ± 1.0	**220.32** ± 121.8	**25.7** ± 3.6	**836.6** ± 368	**646** ± 178
10	**99.7** ± 3.8	**29.7** ± 1.45	-	**18.6** ± 2.9	**1610** ± 310	**1370** ± 287
30	**181.0** ± 11.5	**29.0** ± 1.9	-	**15.4** ± 2.1	**1876** ± 342	**1568** ± 267
60	-	**36.7** ± 0.6	-	**22.2** ± 1.2	**2098** ± 756	**1888** ± 545
